# 4-Methyl-3-phenyl-2,4-dihydro­pyrazolo­[4,3-*c*][1,2]benzothia­zine 5,5-dioxide^1^

**DOI:** 10.1107/S1600536812033284

**Published:** 2012-08-01

**Authors:** Sana Aslam, Hamid Latif Siddiqui, Matloob Ahmad, Muhammad Zia-ur-Rehman, Masood Parvez

**Affiliations:** a Institute of Chemistry, University of the Punjab, Lahore 54590, Pakistan; b Department of Chemistry, Government College University, Faisalabad 38000, Pakistan; c Applied Chemistry Research Centre, PCSIR Laboratories Complex, Lahore 54600, Pakistan; d Department of Chemistry, The University of Calgary, 2500 University Drive NW, Calgary, Alberta, Canada T2N 1N4

## Abstract

In the title mol­ecule, C_16_H_13_N_3_O_2_S, the heterocyclic thia­zine ring adopts a twist chair conformation with the S atom and an adjacent C atom displaced by 0.946 (5) and 0.405 (6) Å, respectively, on the same side of the mean plane formed by the remaining ring atoms. The mean planes of the benzene rings make dihedral angles of 16.61 (10) and 15.32 (10)° with the mean plane of the pyrazole ring. The mol­ecular structure is consolidated by intra­molecular C—H⋯N inter­actions and the crystal packing is stabilized by N—H⋯O and C—H⋯N hydrogen bonds. The crystal studied was an inversion twin with the refined ratio of the twin components being 0.53 (11):0.47 (11).

## Related literature

For the biological activity of related compounds, see: Turck *et al.* (1996 ▶); Silverstein *et al.* (2000 ▶); Lombardino *et al.* (1973 ▶); Zinnes *et al.* (1973 ▶); Ahmad *et al.* (2010*a* ▶,*b* ▶). For related structures, see: Siddiqui *et al.* (2008 ▶, 2009 ▶).
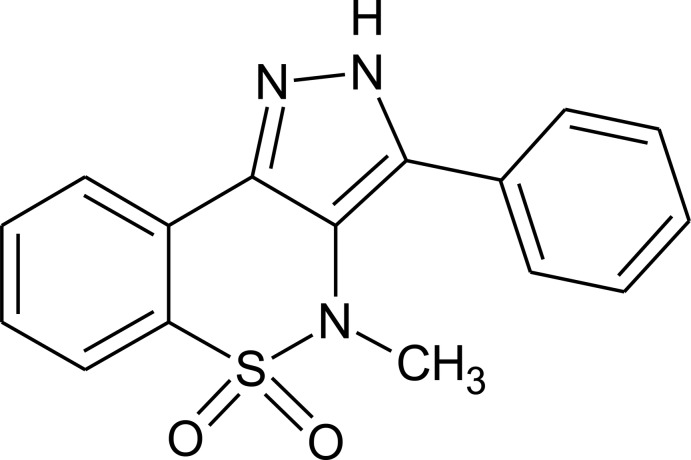


## Experimental

### 

#### Crystal data

C_16_H_13_N_3_O_2_S
*M* *_r_* = 311.35
Orthorhombic, 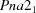

*a* = 12.1028 (5) Å
*b* = 16.3934 (7) Å
*c* = 7.0962 (3) Å
*V* = 1407.93 (10) Å^3^
*Z* = 4
Mo *K*α radiation
μ = 0.24 mm^−1^
*T* = 295 K
0.20 × 0.10 × 0.06 mm

#### Data collection

Nonius KappaCCD diffractometer
Absorption correction: multi-scan (*SORTAV*; Blessing, 1997 ▶) *T*_min_ = 0.953, *T*_max_ = 0.986
8451 measured reflections
3037 independent reflections
2798 reflections with *I* > 2σ(*I*)
*R*_int_ = 0.049

#### Refinement

*R*[*F* ^2^ > 2σ(*F* ^2^)] = 0.049
*wR*(*F* ^2^) = 0.112
*S* = 1.08
3037 reflections
201 parameters
1 restraint
H-atom parameters constrained
Δρ_max_ = 0.20 e Å^−3^
Δρ_min_ = −0.24 e Å^−3^
Absolute structure: inversion twin; used 1306 unmerged Friedel pairs (Flack, 1983 ▶)
Flack parameter: 0.53 (11)

### 

Data collection: *COLLECT* (Nonius, 1999 ▶); cell refinement: *DENZO* (Otwinowski & Minor, 1997 ▶); data reduction: *SCALEPACK* (Otwinowski & Minor, 1997 ▶); program(s) used to solve structure: *SHELXS97* (Sheldrick, 2008 ▶); program(s) used to refine structure: *SHELXL97* (Sheldrick, 2008 ▶); molecular graphics: *ORTEP-3 for Windows* (Farrugia, 1997 ▶); software used to prepare material for publication: *SHELXL97*.

## Supplementary Material

Crystal structure: contains datablock(s) global, I. DOI: 10.1107/S1600536812033284/yk2066sup1.cif

Structure factors: contains datablock(s) I. DOI: 10.1107/S1600536812033284/yk2066Isup2.hkl

Supplementary material file. DOI: 10.1107/S1600536812033284/yk2066Isup3.cml

Additional supplementary materials:  crystallographic information; 3D view; checkCIF report

## Figures and Tables

**Table 1:** Hydrogen-bond geometry (Å, °)

*D*—H⋯*A*	*D*—H	H⋯*A*	*D*⋯*A*	*D*—H⋯*A*
N2—H2*N*⋯O2^i^	0.86	2.12	2.912 (3)	152
C3—H3⋯N3^ii^	0.93	2.56	3.429 (4)	155
C16—H16⋯N1	0.93	2.62	3.263 (4)	127

Symmetry codes: (i) 
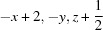
; (ii) 
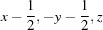
.

**Fig. 1.**
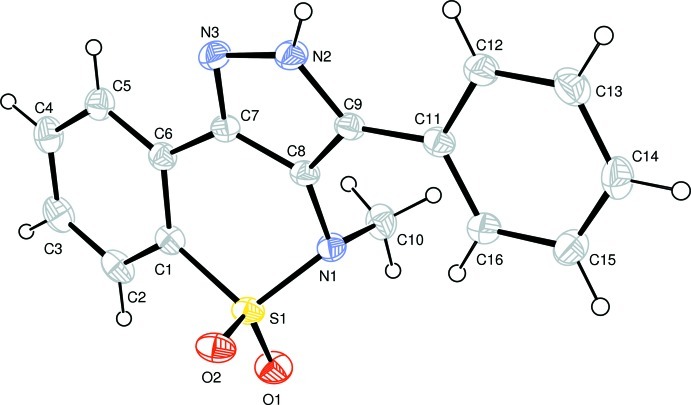
The molecular structure of the title compound with the atom numbering scheme. Displacement ellipsoids are drawn at the 30% probability level. H atoms are presented as small spheres of arbitrary radius.

**Fig. 2.**
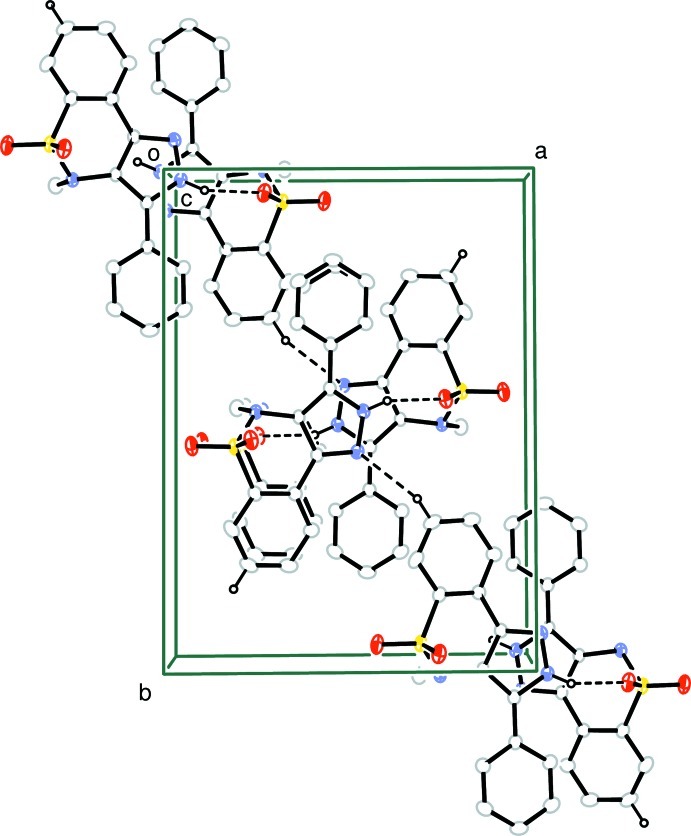
A view of the hydrogen bonding interactions (dotted lines) in the crystal structure of the title compound. H atoms non-participating in hydrogen-bonding are omitted for clarity.

## supplementary crystallographic information

### Comment

Among the broad class of heterocyclic compounds, pyrazole and benzothiazine nuclei are well known for their biological activity potential. Oxicam drugs are benzothiazine based potent anti-inflammatory and analgesic drugs (Turck *et al.* 1996; Lombardino *et al.*, 1973; Zinnes *et al.*, 1973), whereas celecoxib, an anti-inflammatory drug and selective inhibitor of cox-2 enzyme, contains pyrazole fragment (Silverstein *et al.*, 2000). Keeping in view these facts and figures, we have prepared some pyrazolobenzothiazines which contain both of these medicinally important heterocycles fused with eachother (Ahmad *et al.*, 2010**a* & b*). We report here the crystal structure of the title compound.

The bond distances and angles in the title compound (Fig. 1) agree very well with the corresponding bond distances and angles observed in closely related structures (Siddiqui *et al.*, 2008; 2009). The heterocyclic thiazine ring adopts a twist chair conformation with atoms S1 and C1 displaced by 0.946 (5) and 0.405 (6) Å, respectively, on the same side from the mean plane formed by the remaining ring atoms (N1/C6–C8). The mean planes of the benzene rings C1–C6 and C11–C16 make dihedral angles 16.61 (10) and 15.32 (10)°, respectively, with the mean-plane of the pyrazole ring (N2/N3/C7/C8/C10).

The molecular structure of the title compound is consolidated by intramolecular interactions C10—H10C···O1 and C16—H16···N1. The crystal structure is stabilized by intermolecular hydrogen bonding interactions N2—H2N···O2 and C3—H3···N3 (Fig. 2 and Table 1).

### Experimental

A mixture of 3-benzoyl-4-hydroxy-2-methyl-2*H*-1,2-benzothiazine 1,1-dioxide (5.0 g, 0.020 mol), hydrazine hydrate (5 ml) and ethanol (30 ml) was refluxed for 5 h followed by the removal of solvent under vacuum. The residue obtained was washed with cold water to get the title compound as a white crystalline product. Transparent crystals suitable for X-ray crystallographic studies were grown from a CHCl_3_ solution at room temperature by slow evaporation.

### Refinement

All H atoms were positioned geometrically and refined using a riding model, with N—H = 0.86 Å and C—H = 0.93 and 0.96 Å, for aryl and methyl type H-atoms, respectively. The *U*_iso_(H) were allowed at 1.2*U*_eq_(N/C). An absolute structure was not determined as the crystal was a racemic twin with BASF parameter refined to 0.53 (11); 1306 Friedel pairs of reflections were not merged. A low angle reflection (0 1 1) was omitted as it was hindered by the beam stop.

### Crystal data

**Table:**

C_16_H_13_N_3_O_2_S	*F*(000) = 648
*M**_r_* = 311.35	*D*_x_ = 1.469 Mg m^−^^3^
Orthorhombic, *P**n**a*2_1_	Mo *K*α radiation, λ = 0.71073 Å
Hall symbol: P 2c -2n	Cell parameters from 1837 reflections
*a* = 12.1028 (5) Å	θ = 1.0–27.5°
*b* = 16.3934 (7) Å	µ = 0.24 mm^−^^1^
*c* = 7.0962 (3) Å	*T* = 295 K
*V* = 1407.93 (10) Å^3^	Prism, colourless
*Z* = 4	0.20 × 0.10 × 0.06 mm

### Data collection

**Table:**

Nonius KappaCCD diffractometer	3037 independent reflections
Radiation source: fine-focus sealed tube	2798 reflections with *I* > 2σ(*I*)
Graphite monochromator	*R*_int_ = 0.049
ω and φ scans	θ_max_ = 27.5°, θ_min_ = 2.5°
Absorption correction: multi-scan (*SORTAV*; Blessing, 1997)	*h* = −15→15
*T*_min_ = 0.953, *T*_max_ = 0.986	*k* = −21→21
8451 measured reflections	*l* = −9→9

### Refinement

**Table:**

Refinement on *F*^2^	Secondary atom site location: difference Fourier map
Least-squares matrix: full	Hydrogen site location: inferred from neighbouring sites
*R*[*F*^2^ > 2σ(*F*^2^)] = 0.049	H-atom parameters constrained
*wR*(*F*^2^) = 0.112	*w* = 1/[σ^2^(*F*_o_^2^) + (0.0308*P*)^2^ + 1.3942*P*] where *P* = (*F*_o_^2^ + 2*F*_c_^2^)/3
*S* = 1.08	(Δ/σ)_max_ < 0.001
3037 reflections	Δρ_max_ = 0.20 e Å^−^^3^
201 parameters	Δρ_min_ = −0.24 e Å^−^^3^
1 restraint	Absolute structure: racemic twin; used 1306 unmerged Friedel pairs (Flack, 1983)
Primary atom site location: structure-invariant direct methods	Flack parameter: 0.53 (11)

### Special details

**Table:**

Geometry. All s.u.'s (except the s.u. in the dihedral angle between two l.s. planes) are estimated using the full covariance matrix. The cell s.u.'s are taken into account individually in the estimation of s.u.'s in distances, angles and torsion angles; correlations between s.u.'s in cell parameters are only used when they are defined by crystal symmetry. An approximate (isotropic) treatment of cell s.u.'s is used for estimating s.u.'s involving l.s. planes.
Refinement. Refinement of *F*^2^ against ALL reflections. The weighted *R*-factor *wR* and goodness of fit *S* are based on *F*^2^, conventional *R*-factors *R* are based on *F*, with *F* set to zero for negative *F*^2^. The threshold expression of *F*^2^ > σ(*F*^2^) is used only for calculating *R*-factors(gt) *etc*. and is not relevant to the choice of reflections for refinement. *R*-factors based on *F*^2^ are statistically about twice as large as those based on *F*, and *R*- factors based on ALL data will be even larger.

### Fractional atomic coordinates and isotropic or equivalent isotropic displacement parameters (Å^2^)

**Table:**

	*x*	*y*	*z*	*U*_iso_*/*U*_eq_	
S1	0.68932 (5)	−0.05378 (4)	0.08389 (12)	0.03515 (17)	
O1	0.57190 (16)	−0.05177 (14)	0.1024 (4)	0.0499 (6)	
O2	0.73666 (18)	−0.03884 (14)	−0.0982 (3)	0.0431 (5)	
N1	0.74275 (18)	0.01482 (14)	0.2279 (4)	0.0337 (5)	
N2	1.03707 (18)	0.01490 (15)	0.2736 (4)	0.0382 (6)	
H2N	1.1019	0.0358	0.2852	0.046*	
N3	1.0195 (2)	−0.06632 (14)	0.2734 (4)	0.0390 (6)	
C1	0.7415 (2)	−0.14793 (18)	0.1673 (4)	0.0358 (6)	
C2	0.6783 (3)	−0.2185 (2)	0.1532 (5)	0.0440 (8)	
H2	0.6065	−0.2164	0.1068	0.053*	
C3	0.7239 (3)	−0.2915 (2)	0.2091 (5)	0.0495 (8)	
H3	0.6826	−0.3392	0.1997	0.059*	
C4	0.8309 (3)	−0.29478 (19)	0.2793 (5)	0.0478 (8)	
H4	0.8610	−0.3447	0.3148	0.057*	
C5	0.8928 (3)	−0.22450 (18)	0.2968 (5)	0.0408 (7)	
H5	0.9637	−0.2271	0.3471	0.049*	
C6	0.8497 (2)	−0.14990 (17)	0.2397 (4)	0.0332 (6)	
C7	0.9096 (2)	−0.07256 (17)	0.2537 (4)	0.0327 (6)	
C8	0.8602 (2)	0.00418 (16)	0.2414 (4)	0.0307 (6)	
C9	0.9449 (2)	0.06124 (16)	0.2543 (4)	0.0324 (6)	
C10	0.6875 (3)	0.0225 (2)	0.4135 (5)	0.0475 (8)	
H10A	0.7147	0.0700	0.4774	0.057*	
H10B	0.7027	−0.0251	0.4880	0.057*	
H10C	0.6092	0.0275	0.3953	0.057*	
C11	0.9476 (2)	0.15030 (17)	0.2437 (4)	0.0330 (6)	
C12	1.0427 (3)	0.19349 (18)	0.2942 (5)	0.0399 (7)	
H12	1.1040	0.1658	0.3404	0.048*	
C13	1.0454 (3)	0.2779 (2)	0.2753 (5)	0.0473 (8)	
H13	1.1087	0.3064	0.3095	0.057*	
C14	0.9552 (3)	0.31974 (19)	0.2063 (5)	0.0466 (8)	
H14	0.9577	0.3762	0.1935	0.056*	
C15	0.8613 (3)	0.2772 (2)	0.1563 (5)	0.0463 (8)	
H15	0.8002	0.3051	0.1099	0.056*	
C16	0.8575 (3)	0.19346 (19)	0.1748 (5)	0.0405 (7)	
H16	0.7937	0.1655	0.1407	0.049*	

### Atomic displacement parameters (Å^2^)

**Table:**

	*U*^11^	*U*^22^	*U*^33^	*U*^12^	*U*^13^	*U*^23^
S1	0.0238 (3)	0.0454 (4)	0.0363 (3)	−0.0041 (3)	−0.0014 (3)	0.0004 (4)
O1	0.0255 (9)	0.0642 (14)	0.0602 (16)	−0.0059 (9)	−0.0010 (12)	−0.0025 (13)
O2	0.0328 (11)	0.0613 (14)	0.0351 (12)	−0.0066 (10)	−0.0014 (9)	0.0053 (11)
N1	0.0255 (11)	0.0361 (12)	0.0394 (13)	0.0005 (9)	0.0004 (10)	−0.0021 (11)
N2	0.0275 (11)	0.0361 (12)	0.0511 (16)	−0.0026 (10)	−0.0064 (11)	0.0006 (12)
N3	0.0299 (12)	0.0363 (12)	0.0508 (16)	0.0026 (9)	−0.0067 (12)	−0.0013 (12)
C1	0.0328 (14)	0.0401 (16)	0.0343 (15)	−0.0047 (12)	0.0035 (12)	−0.0021 (13)
C2	0.0412 (17)	0.0538 (19)	0.0370 (16)	−0.0166 (14)	0.0052 (14)	−0.0069 (15)
C3	0.060 (2)	0.0394 (17)	0.049 (2)	−0.0164 (15)	0.0029 (17)	−0.0051 (15)
C4	0.062 (2)	0.0345 (15)	0.0468 (19)	−0.0032 (14)	0.0076 (17)	−0.0014 (14)
C5	0.0442 (16)	0.0382 (16)	0.0399 (17)	0.0011 (13)	0.0043 (14)	0.0002 (13)
C6	0.0334 (13)	0.0364 (14)	0.0298 (14)	−0.0009 (11)	0.0019 (12)	−0.0022 (12)
C7	0.0282 (13)	0.0352 (13)	0.0348 (15)	−0.0003 (11)	−0.0022 (12)	−0.0015 (12)
C8	0.0247 (12)	0.0365 (14)	0.0310 (14)	−0.0012 (10)	−0.0027 (11)	−0.0007 (12)
C9	0.0285 (13)	0.0369 (14)	0.0320 (14)	−0.0004 (10)	−0.0045 (12)	−0.0029 (13)
C10	0.0391 (18)	0.0520 (19)	0.051 (2)	0.0004 (14)	0.0098 (15)	−0.0118 (16)
C11	0.0320 (13)	0.0386 (14)	0.0283 (14)	−0.0022 (11)	0.0004 (11)	−0.0007 (12)
C12	0.0375 (15)	0.0390 (15)	0.0433 (18)	−0.0030 (12)	−0.0069 (14)	0.0004 (14)
C13	0.0493 (18)	0.0417 (16)	0.051 (2)	−0.0117 (14)	−0.0067 (16)	−0.0053 (16)
C14	0.061 (2)	0.0300 (14)	0.049 (2)	−0.0025 (14)	0.0047 (16)	−0.0004 (13)
C15	0.0499 (19)	0.0404 (16)	0.0487 (19)	0.0091 (14)	−0.0037 (16)	0.0025 (15)
C16	0.0379 (15)	0.0398 (15)	0.0439 (18)	0.0005 (12)	−0.0069 (14)	−0.0015 (14)

### Geometric parameters (Å, º)

**Table:**

S1—O1	1.428 (2)	C5—H5	0.9300
S1—O2	1.435 (2)	C6—C7	1.464 (4)
S1—N1	1.651 (3)	C7—C8	1.395 (4)
S1—C1	1.770 (3)	C8—C9	1.390 (4)
N1—C8	1.436 (3)	C9—C11	1.462 (4)
N1—C10	1.483 (4)	C10—H10A	0.9600
N2—N3	1.348 (3)	C10—H10B	0.9600
N2—C9	1.356 (3)	C10—H10C	0.9600
N2—H2N	0.8600	C11—C16	1.388 (4)
N3—C7	1.342 (3)	C11—C12	1.398 (4)
C1—C2	1.391 (4)	C12—C13	1.390 (4)
C1—C6	1.406 (4)	C12—H12	0.9300
C2—C3	1.377 (5)	C13—C14	1.379 (5)
C2—H2	0.9300	C13—H13	0.9300
C3—C4	1.389 (5)	C14—C15	1.380 (5)
C3—H3	0.9300	C14—H14	0.9300
C4—C5	1.380 (4)	C15—C16	1.379 (4)
C4—H4	0.9300	C15—H15	0.9300
C5—C6	1.390 (4)	C16—H16	0.9300
			
O1—S1—O2	118.47 (16)	N3—C7—C6	124.3 (2)
O1—S1—N1	108.48 (14)	C8—C7—C6	124.4 (2)
O2—S1—N1	106.54 (13)	C9—C8—C7	106.7 (2)
O1—S1—C1	110.17 (14)	C9—C8—N1	130.7 (2)
O2—S1—C1	107.91 (14)	C7—C8—N1	122.5 (2)
N1—S1—C1	104.31 (14)	N2—C9—C8	103.6 (2)
C8—N1—C10	113.4 (3)	N2—C9—C11	123.1 (2)
C8—N1—S1	110.27 (19)	C8—C9—C11	133.2 (2)
C10—N1—S1	115.5 (2)	N1—C10—H10A	109.5
N3—N2—C9	115.1 (2)	N1—C10—H10B	109.5
N3—N2—H2N	122.5	H10A—C10—H10B	109.5
C9—N2—H2N	122.5	N1—C10—H10C	109.5
C7—N3—N2	103.4 (2)	H10A—C10—H10C	109.5
C2—C1—C6	121.3 (3)	H10B—C10—H10C	109.5
C2—C1—S1	120.3 (2)	C16—C11—C12	118.6 (3)
C6—C1—S1	118.3 (2)	C16—C11—C9	120.6 (3)
C3—C2—C1	118.8 (3)	C12—C11—C9	120.7 (3)
C3—C2—H2	120.6	C13—C12—C11	119.9 (3)
C1—C2—H2	120.6	C13—C12—H12	120.0
C2—C3—C4	120.7 (3)	C11—C12—H12	120.0
C2—C3—H3	119.6	C14—C13—C12	120.7 (3)
C4—C3—H3	119.6	C14—C13—H13	119.6
C5—C4—C3	120.4 (3)	C12—C13—H13	119.6
C5—C4—H4	119.8	C15—C14—C13	119.4 (3)
C3—C4—H4	119.8	C15—C14—H14	120.3
C4—C5—C6	120.3 (3)	C13—C14—H14	120.3
C4—C5—H5	119.9	C16—C15—C14	120.4 (3)
C6—C5—H5	119.9	C16—C15—H15	119.8
C5—C6—C1	118.4 (3)	C14—C15—H15	119.8
C5—C6—C7	123.8 (3)	C15—C16—C11	120.9 (3)
C1—C6—C7	117.8 (3)	C15—C16—H16	119.5
N3—C7—C8	111.2 (2)	C11—C16—H16	119.5
			
O1—S1—N1—C8	168.8 (2)	C5—C6—C7—C8	−163.6 (3)
O2—S1—N1—C8	−62.7 (2)	C1—C6—C7—C8	15.2 (4)
C1—S1—N1—C8	51.3 (2)	N3—C7—C8—C9	0.0 (4)
O1—S1—N1—C10	38.5 (3)	C6—C7—C8—C9	−177.8 (3)
O2—S1—N1—C10	167.1 (2)	N3—C7—C8—N1	−177.3 (3)
C1—S1—N1—C10	−78.9 (2)	C6—C7—C8—N1	4.9 (5)
C9—N2—N3—C7	−0.3 (4)	C10—N1—C8—C9	−86.5 (4)
O1—S1—C1—C2	30.8 (3)	S1—N1—C8—C9	142.1 (3)
O2—S1—C1—C2	−99.9 (3)	C10—N1—C8—C7	90.0 (3)
N1—S1—C1—C2	147.0 (2)	S1—N1—C8—C7	−41.3 (4)
O1—S1—C1—C6	−152.0 (2)	N3—N2—C9—C8	0.3 (4)
O2—S1—C1—C6	77.3 (3)	N3—N2—C9—C11	−177.4 (3)
N1—S1—C1—C6	−35.8 (3)	C7—C8—C9—N2	−0.2 (3)
C6—C1—C2—C3	−0.9 (5)	N1—C8—C9—N2	176.8 (3)
S1—C1—C2—C3	176.2 (3)	C7—C8—C9—C11	177.2 (3)
C1—C2—C3—C4	0.4 (5)	N1—C8—C9—C11	−5.8 (6)
C2—C3—C4—C5	1.0 (6)	N2—C9—C11—C16	162.7 (3)
C3—C4—C5—C6	−1.7 (5)	C8—C9—C11—C16	−14.3 (5)
C4—C5—C6—C1	1.1 (5)	N2—C9—C11—C12	−14.2 (5)
C4—C5—C6—C7	179.8 (3)	C8—C9—C11—C12	168.7 (3)
C2—C1—C6—C5	0.2 (4)	C16—C11—C12—C13	0.1 (5)
S1—C1—C6—C5	−176.9 (2)	C9—C11—C12—C13	177.2 (3)
C2—C1—C6—C7	−178.6 (3)	C11—C12—C13—C14	−0.3 (6)
S1—C1—C6—C7	4.2 (4)	C12—C13—C14—C15	0.2 (6)
N2—N3—C7—C8	0.2 (3)	C13—C14—C15—C16	−0.1 (6)
N2—N3—C7—C6	178.0 (3)	C14—C15—C16—C11	0.0 (5)
C5—C6—C7—N3	18.9 (5)	C12—C11—C16—C15	0.0 (5)
C1—C6—C7—N3	−162.4 (3)	C9—C11—C16—C15	−177.0 (3)

### Hydrogen-bond geometry (Å, º)

**Table:**

*D*—H···*A*	*D*—H	H···*A*	*D*···*A*	*D*—H···*A*
N2—H2*N*···O2^i^	0.86	2.12	2.912 (3)	152
C3—H3···N3^ii^	0.93	2.56	3.429 (4)	155
C10—H10*C*···O1	0.96	2.49	2.883 (4)	104
C16—H16···N1	0.93	2.62	3.263 (4)	127

Symmetry codes: (i) −*x*+2, −*y*, *z*+1/2; (ii) *x*−1/2, −*y*−1/2, *z*.
